# First simultaneous detection of electron and positron bunches at the positron capture section of the SuperKEKB factory

**DOI:** 10.1038/s41598-021-91707-0

**Published:** 2021-06-17

**Authors:** Tsuyoshi Suwada, Muhammad Abdul Rehman, Fusashi Miyahara

**Affiliations:** 1 High Energy Accelerator Research Organization (KEK), Accelerator Laboratory, Tsukuba, Ibaraki 305-0801 Japan; 2 School of High Energy Accelerator Science, The Graduate University for Advanced Studies (SOKENDAI), Tsukuba, Ibaraki 305-0801 Japan

**Keywords:** Applied physics, Particle physics, Techniques and instrumentation

## Abstract

The direct simultaneous detection of electron and positron bunch signals was successfully performed for the first time with wideband pickups and a detection system at the positron capture section of the SuperKEKB factory. The time interval between the electron and positron bunches, their bunch lengths, and bunch intensities depending on the phase of accelerating structures were measured to investigate their capture process and to maximally optimize the positron intensity. The results show that the time intervals were measured in the range of 135–265 ps, and the line-order switch of the electron and positron bunches in the axial direction was clearly observed as a function of the phase. The positron (electron) intensity was maximized at the optimal phase (180$^{\circ }$ shifted from the optimum). These series of measurements have never been experimentally conducted so far. It is demonstrated that the positron intensity can be systematically optimized with this system as functions of beam parameters in multidimensional spaces for any positron capture section.

## Introduction

The SuperKEKB factory^1^ (SKEKB) is a next-generation B-factory that is currently in operation at KEK, after the KEK B-factory^2^ (KEKB) was discontinued in 2010. The SKEKB is an electron ($e^-$)/positron ($e^+$) collider with asymmetric energies; it comprises 4 GeV $e^+$ (LER) and 7 GeV $e^-$ (HER) rings in which the designed stored beam currents are 3.6 A and 2.6 A, respectively. The target luminosity ($8 \times 10^{35}\,\hbox {cm}^{-2}\hbox {s}^{-1}$) of the SKEKB, that is, the rate of $e^-$ and $e^+$ collisions, is 40 times the peak luminosity of the KEKB. The high-energy flavor particle physics experiments^3^, considering the CP violation in B mesons, are the main driver behind this study. To improve the collision rate, the development of not only a low-emittance $e^-$ source but also a powerful and stable $e^+$ source^4–6^ is one of the key elements in this experiment.

The SKEKB injector linac^7^ is an $e^-/e^+$ linear accelerator for the SKEKB; the KEKB injector linac^8^ was upgraded for the abovementioned purpose. The requirements for the injector linac are full energy injection into the SKEKB rings with the $e^-$ and $e^+$ bunch charges of 5 and 4 nC, respectively. The injector linac should deliver low-emittance and high-current $e^-$ and $e^+$ beams to the SKEKB rings. The $e^-$ beam is generated using a new photocathode radio-frequency (rf) gun^9^. On the other hand, the $e^+$ beam is generated by bombarding a tungsten target with high-energy primary electrons with an energy of 3.5 GeV and charges of 10 nC. The positrons are to be efficiently captured using a new flux concentrator and large-aperture S-band accelerating structures^10^ in the $e^+$ capture section, and they are to be damped to the level required for the low-emittance beam through a new damping ring (DR)^11^.

Since both the electrons and positrons with approximately equivalent amounts of bunch charges are generated at the target, not only the positrons but also the electrons are simultaneously captured and accelerated (or decelerated) in the capture section. Note that both the electrons and positrons pass synchronously through the capture section with a certain time interval that is dependent on the operation condition of the capture section. The time interval between the $e^-$ and $e^+$ bunches is very short with a time range from 135 to 265 ps, which is dependent on the capture phase of accelerating structures.

The time interval between the $e^-$ and $e^+$ bunches, bunch lengths, and bunch intensities for each $e^-$ and $e^+$ bunch are very important parameters that can be fundamentally investigated on the basis of detailed beam dynamics at the capture section. However, they have never been measured because the time interval is too short to detect them independently, while they are generally simulated on the basis of beam dynamics in multidimensional transverse and longitudinal phase spaces. Thus, it is a challenging to experimentally verify and elucidate complicated beam dynamics for both positrons and electrons in the capture section in order to fully understand them and to maximize the positron intensity under an optimized operation condition.

For this purpose, new beam monitors with wideband pickups and a detection system were installed at the capture section to independently detect $e^-$ and $e^+$ signals during the summer shutdown of 2019. They are essential diagnostic instruments to fully investigate the $e^-$ and $e^+$ capture process and to maximally optimize the $e^+$ intensity. Both the electrons and positrons generated at the target are formed into their steady bunched beams through their phase slip process (called the capture process) in accelerating structures of the capture section. Thus, for example, the time interval between the $e^-$ and $e^+$ bunches is one of the important beam parameters that are strongly dependent on the capture phase of the accelerating structures. In conventional operation, a $e^+$ bunch intensity can be normally optimized by measuring it using a conventional beam intensity monitor installed after an $e^-$ and $e^+$ bunch separator (called chicane).

Multidimensional optimization schemes should be applied to the capture section to experimentally increase the $e^+$ bunch intensity in a multidimensional-parameter space, which is based on electromagnetic fields of accelerating structures (capture phases) and magnetic solenoid and dipole fields in the capture section, and transverse positions, angles, and radius of the primary electrons impinging on the target. It is generally difficult to find out not the local optimum but the true global optimum in such a multidimensional optimization scheme. This is because it is difficult to directly measure independent $e^-$ and $e^+$ bunches by a conventional technique. This is the reason why new beam monitors should be installed in the capture section.

## Results

### Transient responses of pickup signals

**Figure 1:**
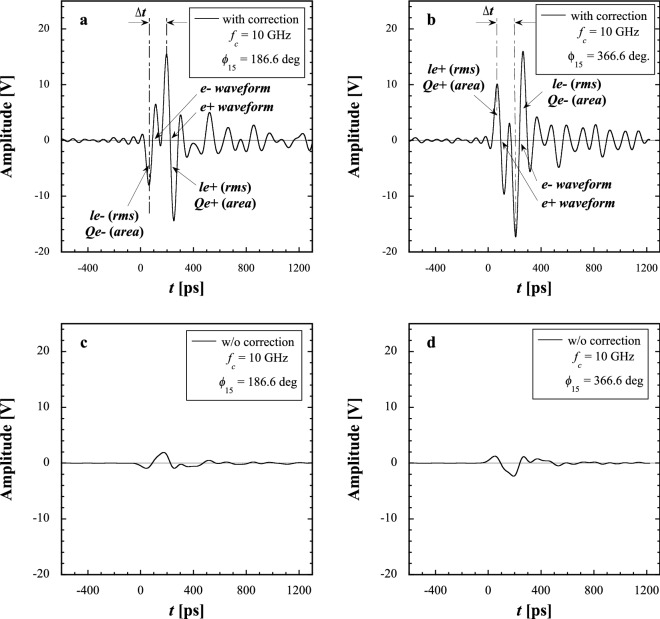
Secondary-generated $e^-$ and $e^+$ signal waveforms measured using the new beam monitor in the $e^+$ mode, (**a**) with and (**c**) without a transmission cable-loss correction at the capture phase $\phi _{15}=186.6^{\circ }$, and (**b**) with and (**d**) without that at the capture phase $\phi _{15}=366.6^{\circ }$. $f_c$ was fixed to 10 GHz.

The frequency characteristics of the pickup signals detected by the new beam monitor were measured to investigate the transient responses of the pickups along with signal transmission cables for the injection $e^-$ and $e^+$ modes as a function of the cutoff frequency ($f_c$) of a wideband oscilloscope. Figure 1a–d show typical signal waveforms measured using the new monitor in the $e^+$ mode with and without transmission cable-loss correction at the capture phase $\phi _{15}=186.6^{\circ }$ (see Fig. 1a,c, respectively), and with and without that at the capture phase $\phi _{15}=366.6^{\circ }$ (see Fig. 1b,d, respectively). $f_c$ was fixed to 10 GHz.

The pickup signal is a bipolar signal obtained by differentiating the envelope of a bunched beam by time. It indicates two successive bipolar signals for both secondary-generated $e^-$ and $e^+$ bunches simultaneously detected at the capture section. It can be found that the signals with corrections using cable-loss data (called corrected signals) show that the transient responses are properly corrected (see Fig. 1a,b). On the other hand, the transient responses of the raw signals without any corrections are too slow owing to high-frequency cable losses to clearly identify both the $e^-$ and $e^+$ signals independently despite any cutoff frequencies (see Fig. 1c,d). Thus, the detected raw signals must be corrected. The oscilloscope can automatically correct them in the frequency domain and display the corrected signals in real time in the time domain pulse by pulse without any difficulty.

It is clearly found that the corrected signal shows that the first and second bipolar signals correspond to the $e^-$ and $e^+$ bunch signals in temporal order, respectively, because the polarity of the signal waveform depends on the charge sign of each bunch (see Fig. 1a). It is also clear that the first and second bipolar signals correspond to the $e^+$ and $e^-$ bunch signals in temporal order, respectively (see Fig. 1b). It is understood that the corrected signals with a bandwidth of 10 GHz show clear separation for the $e^-$ and $e^+$ bunch signals, which is described in detail in the next section. On the other hand, it is very difficult to independently separate the $e^-$ and $e^+$ bunch signals in the raw signals owing to the large signal distortion caused by transmission cable losses (see Fig. 1c,d). Thus, the cutoff frequency set to the oscilloscope is one of the important parameters in this measurement system.

### Frequency characteristics of beam parameters

Beam parameters extracted from the signal waveform were analyzed in this measurement. They are the time interval $\Delta t$ between successive $e^-$ and $e^+$ bunches, their bunch lengths in root mean square (rms), $l_{e^-}$ and $l_{e^+}$, and their bunch intensities, $Q_{e^-}$ and $Q_{e^+}$, in the $e^+$ mode. The bunch yields are similarly defined as the ratio of the intensity of the secondary-generated bunch to that of the primary electron bunch, $Q_{pe^-}$. The $e^-$ and $e^+$ bunch yields are represented by $Y_{e^-}\equiv Q_{e^-}/Q_{pe^-}$ and $Y_{e^+}\equiv Q_{e^+}/Q_{pe^-}$, respectively. Note that the bunch yields are more beneficial than the bunch intensities because the bunch yield indicates the generation efficiency of the secondary-generated bunch.

**Figure 2:**
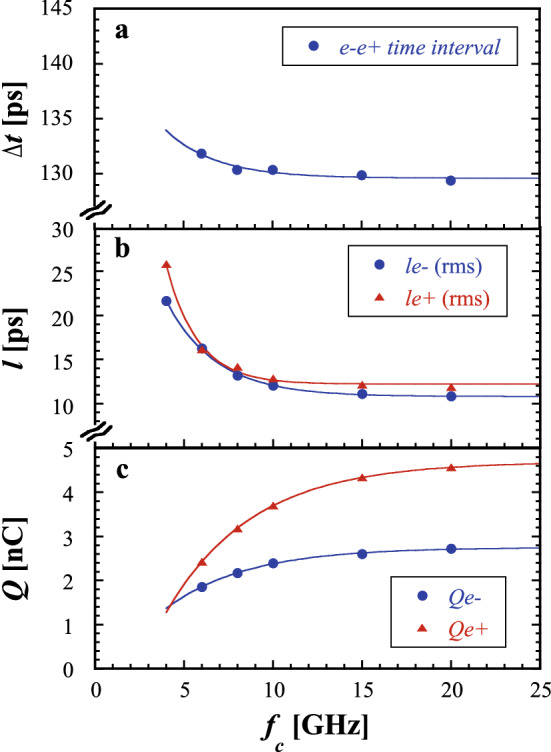
Frequency characteristics measured under the nominal operation condition of the $e^+$ mode as a function of the cutoff frequency for (**a**) the time interval between the $e^-$ and $e^+$ bunches, (**b**) the $e^-$ and $e^+$ bunch lengths, and (**c**) the $e^-$ and $e^+$ bunch intensities. The solid lines show fitting curves of the data using an exponential function.

Figure 2a–c show the frequency characteristics of the time interval, the bunch lengths, and intensities of the $e^-$ and $e^+$ bunches with corrections, respectively, as a function of the cutoff frequency under the nominal operation condition.

Each beam parameter approaches to each asymptotically convergent point in the frequency domain. Each solid line shows a fitting curve of the data using an exponential function. It is found that the convergent frequency points, that is, the optimized cutoff frequencies, are different with different beam parameters and also for the $e^-$ and $e^+$ bunches. These are analyzed to be $\sim$ 20 GHz, $\sim$ 15 GHz, and $\sim$ 25 GHz in the measurements of the time interval, bunch lengths, and bunch intensities in the $e^+$ mode, respectively. Note that although the proper cutoff frequency should be set to greater than 10 GHz, higher-order TE modes may be generated in 10D coaxial cables of the transmission line^12^, and the measurement accuracy of the beam parameters may be restricted because the signal waveforms measured in the time domain are deformed in the high-frequency region that is discussed in detail in “Methods”. Thus, the cutoff frequency was set to $f_c=10$ GHz for the oscilloscope in the following measurements. Although the beam parameters do not converge sufficiently to each asymptotically convergent point owing to the insufficient transient response of the corrected signals, the difference of the analyzed beam parameter to each asymptotic value at each cutoff frequency was handled as one of the systematic errors.

The definition of each beam parameter is clearly specified here. The time interval is defined by a time difference between the peak point of the first pulsed lobe for the $e^+$-bunch bipolar signal and that for the $e^-$-bunch bipolar signal. Since the first and second bipolar signals under the nominal operation condition of the $e^+$ mode correspond to those of the $e^-$ and $e^+$ bunches, respectively, the time interval is defined as $\Delta t > 0$ (see Fig. 1a). On the other hand, since the first and second bipolar signals shown in Fig. 1b correspond to those of the $e^+$ and $e^-$ bunches, respectively, the time interval is defined as $\Delta t < 0$. The bunch lengths are analyzed on the basis of the rms pulse-width calculation of the negative (positive) pulsed lobe for the first $e^-$($e^+$)-bunch and second $e^+$($e^-$)-bunch bipolar signals (see Fig. 1a,b). The bunch intensities are calculated by a summation of four pickup signals, where the pulse areas of the negative (positive) pulsed lobe for the first $e^-$($e^+$)-bunch and second $e^+$($e^-$)-bunch bipolar signals are separately analyzed for the $e^-$ and $e^+$ bunches (see Fig. 1a,b).

Calibrations for the bunch intensity were carried out using the charges measured by a beam position monitor (BPM, SP16_57) for an injection $e^-$ bunch with bunch charges of $\sim$2 nC rather than the use of the $e^+$ bunch because the obtained signal is one bipolar signal, which effectively reduces the effect of the transient response compared with two successive bipolar signals. The first calibration procedure was carried out at $f_c=10$ GHz; however, it is apparent that the bandwidth for the intensity calibration procedure is insufficient (see Fig. 2c). Thus, the second calibration procedure was carried out, which was based on corrections to the first calibration procedure. The correction factors for the secondary-generated $e^-$ and $e^+$ bunches were analyzed to further correct the difference between the bunch intensities at 10 GHz and those at 20 GHz. Thus, the difference between each asymptotic value analyzed at $f_c=25$ GHz and those at 20 GHz was handled as one of the systematic errors.

### Error analyses of beam parameters

The measurement errors were estimated on the basis of both statistical and systematic error analyses. In the time-interval and bunch-length measurements, data were obtained by taking averages of the four pickups, and their statistical errors were defined by calculating standard errors. On the other hand, the systematic errors ($\sigma ^{sys}$) were analyzed on the basis of the frequency characteristics of the signal waveforms while taking into account the transient responses.

Note that there is another systematic error ($\sigma ^{wake}$) based on wakefield effects, which is different from that based on the frequency characteristics. When a charged particle travels across an accelerator structure, it induces electromagnetic fields (called wakefields^13^) that are left mainly behind the generating particle. These electromagnetic fields act back on the beam and affect its motion in both the transverse and longitudinal directions, which are called wakefield effects.

In this measurement, the wakefields of a first bunch act back on a second bunch and affect its motion. As shown in Fig. 1a, in this measurement, the $e^-$ bunch is in front and the $e^+$ bunch is in the rear. It can be seen that after the $e^+$ signal, a subsequent ringing waveform exists during a time span of more than 1000 ps in the corrected signal. This ringing signal may be caused by wakefields of not only the front $e^-$ bunch but also the rear $e^+$ bunch. It is, however, difficult to quantitatively estimate and remove the subsequent ringing signal from the corrected signal, because the frequency spectrum of wakefields exists just inside that of the corrected signal in the frequency domain. The detailed analysis of the wakefield effects is beyond the scope of the present paper.

Thus, simple characteristic analyses in the time domain are performed without any filtering procedures to estimate systematic errors caused by wakefield effects. First, it is assumed that the subsequent ringing signal just after the rear bunch is caused by wakefields of both the front and rear bunches, and then the main corrected signal waveform without any ringing signal (that is, main bipolar signal) is separated from the ringing signal, although the main bipolar signal itself may be affected by the wakefield effects. Then, the main corrected signal is time-shifted by a small time step, and the shifted main corrected signal is simply superimposed on the ringing signal. By such procedures, the main corrected signal may be deformed owing to the deformation of the signal waveform superimposed with the ringing signal in the time domain. Secondly, the beam parameters are similarly analyzed. They may spread to some extent around the original beam parameters if they are similarly analyzed after repeating a successive time step one after another in the time domain. Systematic errors due to the wakefield effects can be estimated on the basis of the corresponding beam parameter analyses. Finally, total errors ($\sigma ^{tot}$) are obtained by calculating the root-mean-square sum for both statistical and systematic errors based on the analyses in the frequency characteristics and wakefield effects. The systematic errors analyzed under the nominal operation condition are summarized in Table 1.

**Table 1:** Summary of the systematic errors for the beam parameters analyzed under the nominal operation condition of the $e^{+}$ mode.

Parameters	1st and 2nd bunches ($e^{-}e^{+}$)			1st bunch ($e^{-}$)			2nd bunch ($e^{+}$)		
	$\sigma ^{sys}_{\Delta t}$	$\sigma ^{wake}_{\Delta t}$	$\sigma ^{tot}_{\Delta t}$	$\sigma ^{sys}$	$\sigma ^{wake}$	$\sigma ^{tot}$	$\sigma ^{sys}$	$\sigma ^{wake}$	$\sigma ^{tot}$
Time interval (ps)	0.5	4	4						
Bunch length (ps)				1	0.8	1	0.5	0.4	0.7
Bunch yield				0.01	0.04	0.04	0.02	0.04	0.05

### Analyses of beam parameters

**Figure 3:**
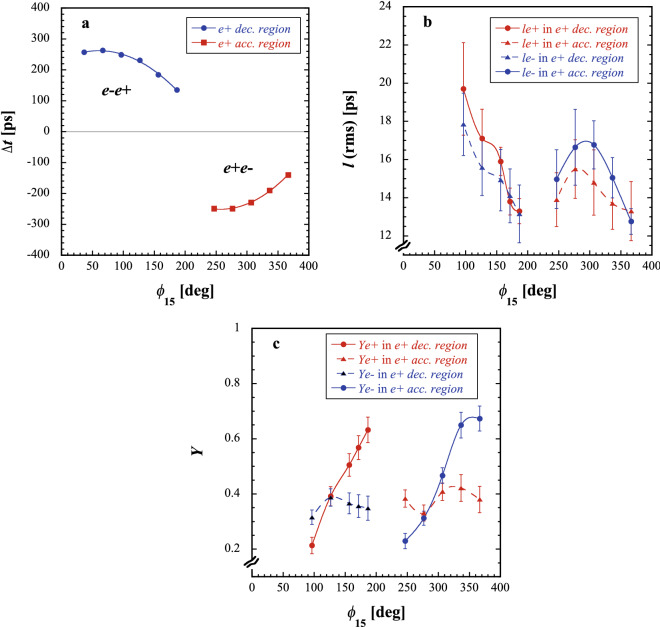
Variations in the measured beam parameters as a function of the capture phase $\phi _{15}$, (**a**) in the time-interval measurement, (**b**) in the bunch-length measurement, and (**c**) in the bunch-yield measurement. The cutoff frequency was fixed to $f_c=10$ GHz.

Figure 3a–c show the variations in the beam parameters, the time interval between the $e^-$ and $e^+$ bunches, the $e^-$ and $e^+$ bunch lengths (rms), and the $e^-$ and $e^+$ bunch yields as a function of the capture phase $\phi _{15}$, respectively. The other parameters in the $e^+$ capture section were set to the nominal operation ones in the $e^+$ mode. The nominal capture phase is $\phi _{15}=186.6^{\circ }$, whereas the capture phase $\phi _{16}$ was fixed to the nominal one during the measurements.

It is clear that there are two regions in the time-interval measurement as a function of the phase $\phi _{15}$, as shown in Fig. 3a. The phase region of $30\lesssim \phi _{15}\lesssim 190^{\circ }$ indicates that the $e^-$ bunch exists ahead of the $e^+$ bunch, which is in the $e^-$ acceleration region (or equivalent $e^+$ deceleration region). On the other hand, the phase region of $240 \lesssim \phi _{15} \lesssim 370^{\circ }$ indicates that the $e^+$ bunch exists ahead of the $e^-$ bunch, which is in the $e^-$ deceleration region (or equivalent $e^+$ acceleration region). The time intervals between the two bunches vary in the time region of about 135–265 ps in the $e^+$ deceleration region and that of about −140 to − 250 ps in the $e^+$ acceleration region, depending on the capture phase $\phi _{15}$. The variations in the time-interval measurement show clear symmetrical behaviors depending on the capture phase $\phi _{15}$.

The variations in the $e^-$ and $e^+$ bunch-length measurement depending on the capture phase $\phi _{15}$ are shown in Fig. 3b. In the $e^+$ deceleration region, the $e^+$ bunch length is slightly larger than the $e^-$ bunch length; however, this relationship is reversed in the $e^+$ acceleration region. It is interesting that the bunch length of the $e^-$ coincides with that of the $e^+$ at the intersection point for these variation curves. At the intersection point, their bunch lengths are obtained to be equal and almost minimum. Although the results are mainly dominated by beam dynamics, they indicate that both electrons and positrons are approximately symmetrically formed into their steady bunched beams through each phase slip process in the accelerating structures of the capture section under the nominal operation condition of the $e^+$ mode.

It is clear that the $e^+$ yield is maximized at the nominal operation phase ($\phi _{15}=186.6^{\circ }$), as shown in Fig. 3c, which is in the $e^+$ deceleration region. On the other hand, the $e^-$ yield is maximized at the phase $\phi _{15}=366.6^{\circ }$, which is in the $e^+$ acceleration region and shifted $180^{\circ }$ from the phase giving the maximal $e^+$ yield. This result shows that the maximal $e^+$ ($e^-$) yield is obtained not in the $e^+$ acceleration (deceleration) region, but in the $e^+$ deceleration (acceleration) region under the nominal operation condition.

It is found that the time interval and the bunch lengths of both the electrons and positrons are almost minimum at the phase $\phi _{15}$ giving the maximal $e^+$ yield. It is also interesting that the maximal $e^-$ yield at the phase $\phi _{15}=366.6^{\circ }$ is $\sim$6 % larger than that of the $e^+$ yield. This is caused by the Compton effect^14^ dominated in the secondary $e^-$ generation through electromagnetic interaction in the target in comparison with the secondary $e^+$ generation. It was also verified that the $e^+$ yield was $\sim$ 0.63 under the nominal operation condition at the capture section, whereas it was obtained as $\sim$ 0.45 on average at the BPM location (SP16$\_$57) after the chicane. This result shows that the beam loss at the capture section is quantitatively estimated as $\sim$ 29%.

The beam parameters for the first ($e^-$) and second ($e^+$) bunches obtained under the nominal operation condition of the $e^+$ mode are summarized in Table 2.

**Table 2:** Summary of the beam parameters for the first ($e^-$) and second ($e^+$) bunches obtained under the nominal operation condition of the $e^+$ mode.

Params.	1st and 2nd bunches ($e^{-}e^{+}$)	1st bunch ($e^{-}$)	2nd bunch ($e^{+}$)
$\Delta t$ (ps)	$135\pm 4$		
*l* (ps)		$13\pm 1$	$13.3\pm 0.7$
*Y*		$0.35\pm 0.04$	$0.63\pm 0.05$

These results were successfully obtained for the first time, and they could greatly help in the design of the next-generation $e^+$ source and the development of simulations for the $e^+$ capture section.

## Discussion

The direct simultaneous detection of $e^-$ and $e^+$ bunches was successfully carried out for the first time with wideband pickups and a detection system at the $e^+$ capture section of the SuperKEKB factory. After investigating the frequency characteristics of the detection system and the systematic error analyses for wakefield effects, several beam parameters, the time interval between the $e^-$ and $e^+$ bunches, the $e^-$ and $e^+$ bunch lengths (rms), and the bunch yields were clearly obtained under the nominal operation condition within sufficiently small experimental errors. It was verified that under the nominal operation condition, the $e^+$ yield was optimized to be maximum. It was also found that the maximal $e^+$ yield was $0.63\pm 0.05$ under the nominal operation condition, whereas the maximal $e^-$ yield was $0.67\pm 0.05$ at the capture phase that was shifted $180^{\circ }$ from the phase giving the maximal $e^+$ yield. It is also interesting that the optimized bunch yields for the $e^-$ is $\sim$ 6% larger than that for the $e^+$ owing to the Compton effect. It is of great benefit to apply such a detection system to wideband beam monitors installed in any high-intensity $e^+$ source required for next-generation high-energy accelerator complexes.

## Methods

### Positron source and its capture section of the SuperKEKB factory

**Figure 4:**
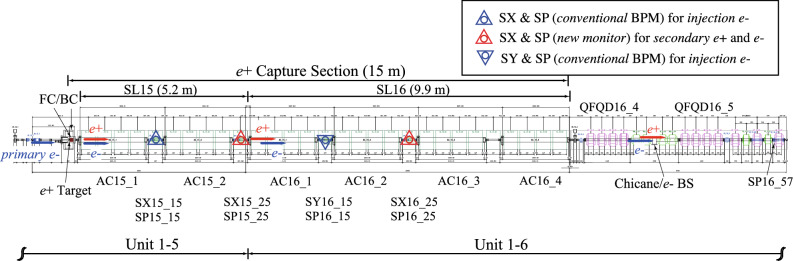
Layout of the positron source and its capture section at the SKEKB injector linac.

The new positron source along with its capture section is briefly described here^15^ and is shown in Fig. 4.

High-current positrons are generated by bombarding a tungsten target with high-energy primary electrons with an energy of 3.5 GeV and charges of 10 nC/bunch for two successive bunches at a repetition rate of 50 Hz at maximum, which can be delivered from a conventional thermionic $e^{-}$ gun. An off-axis $e^+$-production target ($e^{+}$ target) with the use of 14-mm-thick tungsten was designed and built at the middle of sector 1 (unit 1–5). The off-axis target means that injection electrons pass through a hole of 2 mm diameter at the target center, while high-current primary electrons for $e^+$ generation hit the target at 3.5 mm off-axis in the vertical direction from the target center^16^. The primary and injection electrons can be switched by controlling the beam optics pulse by pulse with pulsed steering dipoles and quadrupole magnets installed in front of the target^15^.

The generated positrons must be efficiently captured at the 15-m-long $e^+$ capture section. Immediately following the target, the capture section is located at units 1–5 and 1–6, which comprise rf accelerating structures and magnetic matching devices. The rf accelerating structures comprise two 2-m-long large-aperture S-band accelerating structures^10^ (LAS, energy gain 14–20 MV/m) in unit 1–5 (AC15$\_$1 and $15\_$2) and four 2-m-long LAS (energy gain 10 MV/m) in unit 1–6 (AC16$\_$1, $16\_$2, $16\_$3 and $16\_$4) and they are powered by each high-power klystron with a SLED-type pulse compression system to boost the energy of the captured positrons to 120 MeV on average^7^.

The large transverse emittances of the $e^+$ bunch emerging from the target are transformed to match the capture section aperture with its $\sim$ 0.5 T DC solenoids (SL, 0.4 T and 0.5 T in units 1–5 and 1–6, respectively) by a pseudoadiabatically changing solenoidal field consisting of a 3.5 T pulsed peak field from a flux concentrator (FC) as a strong $e^+$-focusing solenoid with a large energy acceptance^17^. A bridge coil (BC) with a 1.5 T DC solenoidal field is also installed between the FC and downstream DC SLs to make the solenoidal field distribution smooth. The DC solenoidal field exists along the capture section.

A $e^{+}$ bunch conventionally can be first separated from an $e^{-}$ bunch at the chicane composed of four successive bending magnets. Quadrupole focusing and defocusing (QF and QD, respectively) magnetic systems (QFQD16$\_$4 and 16$\_$5) are installed at both ends of the chicane. The electrons are stopped at the center of the chicane by a beam stopper (BS). Transverse bunch positions and the $e^{+}$ bunch intensity normally can be first measured with a BPM (SP16$\_$57) located at the end of unit 1–6 after the chicane, since there are no beam monitors in the capture section. The conversion factor defined by a number of positrons generated per primary $e^{-}$ (called $e^{+}$ yield) was experimentally investigated using this BPM to be $\sim$0.5 under the nominal operation condition. The more detailed designs are described elsewhere^15^.

### New beam monitors with wideband pickups

**Figure 5:**
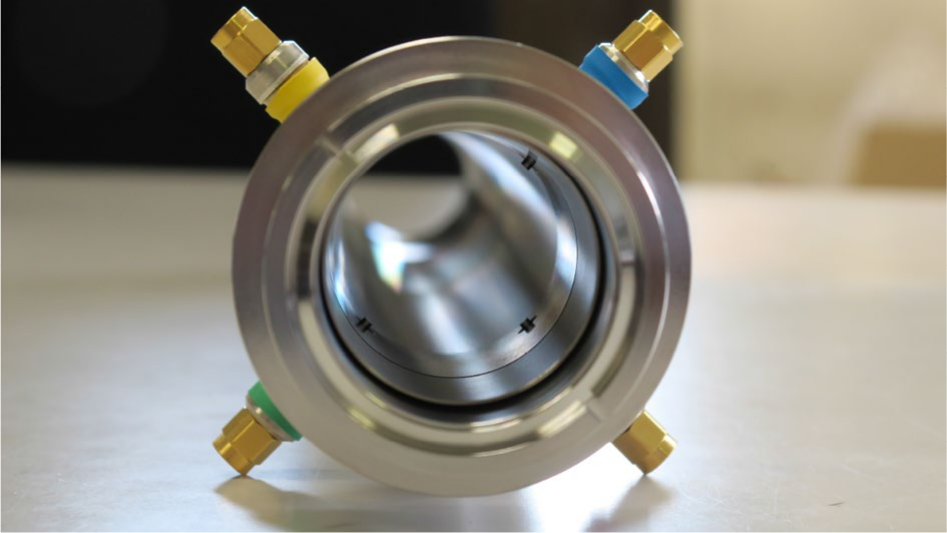
Photograph of the new beam monitor for detecting both electrons and positrons in the $e^{+}$ mode.

A photograph of the new beam monitor is shown in Fig. 5.

The total length of the monitor including two bellows and quick-release flange couplings (NW40, standard KF flange^18^) at both ends is 431 mm, and the inner diameter is 38 mm. The inner surface of the front bellows is covered with a cylindrical pipe to remove the irregularity in order to suppress any wakefield effects caused at the monitor front as much as possible. The pickups of the monitor are made of SMA-type vacuum feedthroughs composed of a central conductor pin made of Kovar and a dielectric substance made of ceramic. The four pickups, two horizontal and two vertical, are mounted on the upstream side of the monitor with $\pi /2$ rotational symmetry. The tips of the center pins protrude for a length of 1 mm toward the monitor center from the inner surface of the monitor. The new monitors were installed at two locations at the center of units 1–5 and 1–6 in the capture section after inserting the new monitor into a cylindrical frame along with a steering magnet.

The pickups have good frequency characteristics, which were tested in the frequency region greater than 10 GHz. The signals detected by the four pickups are directly sent to a wideband real-time oscilloscope (maximal bandwidth 33 GHz, maximal sampling rate 128 GS/s, Keysight Technologies, Inc.^19^) with several coaxial cables, namely, a 2-m-long semirigid coaxial cable, a 15-m-long 10D coaxial cable (10D-HFB-CE, Fujikura Dia Cable, Ltd.^20^), and a 2-m-long RG223 coaxial cable, connected in series. The ends of the coaxial cables are independently connected to four input channels of the oscilloscope with a fixed attenuator of 10 dB in front of each input channel. The four signal waveforms are measured pulse by pulse under the proper measurement condition both with and without corrections on the transmission cable loss. The high-frequency losses of all the coaxial cables were measured by a vector network analyzer (bandwidth 13.5 GHz, Keysight Technologies, Inc.^21^) in advance.

Four pairs of a new beam monitor (SP) and a transverse steering magnet (dipole magnet for correcting a beam orbit in the transverse direction) were installed in a limited space between the SLs at different locations of units 1–5 and 1–6 along the beam line (see Fig. 4). The steering magnets comprise three horizontal (*x*) magnets and one vertical (*y*) magnet, SXs and SY, respectively. Two SPs with conventional stripline electrodes (SP15$\_$15 and SP16$\_$15) were installed for measuring the injection electrons; however, the other new SPs (SP15$\_$25 and SP16$\_$25) were installed for measuring the secondary-generated electrons and positrons in the $e^{+}$ mode in order to independently measure the time interval between the $e^{-}$ and $e^{+}$ bunches, their bunch lengths, and bunch intensities, and also their transverse beam positions, pulse by pulse.
